# Self-Selected Versus Assigned Target to Reduce Smartphone Use and Improve Mental Health: Protocol for a Randomized Controlled Trial

**DOI:** 10.2196/53756

**Published:** 2024-05-06

**Authors:** Kamal Kant Sharma, Jeeva Somasundaram, Ashish Sachdeva

**Affiliations:** 1 Max Institute of Healthcare Management Indian School of Business Sahibzada Ajit Singh Nagar, Punjab India; 2 IE Business School Madrid Spain

**Keywords:** screen time, monetary incentives, target selection, mental health, mobile phone

## Abstract

**Background:**

Smartphones have become integral to people’s lives, with a noticeable increase in the average screen time, both on a global scale and, notably, in India. Existing research links mobile consumption to sleep problems, poor physical and mental health, and lower subjective well-being. The comparative effectiveness of monetary incentives given for self-selected versus assigned targets on reducing screen time and thereby improving mental health remains unanswered.

**Objective:**

This study aims to assess the impact of monetary incentives and target selection on mobile screen time reduction and mental health.

**Methods:**

We designed a 3-armed randomized controlled trial conducted with employees and students at an educational institution in India. The study is conducted digitally over 12 weeks, including baseline (2 weeks), randomization (1 week), intervention (5 weeks), and postintervention (4 week) periods. We emailed the employees and students to inquire about their interest in participation. Those who expressed interest received detailed study information and consent forms. After securing consent, participants were asked to complete the initial survey and provide their mobile screen time during the baseline period. At the beginning of the intervention period, the participants were randomly allocated into 1 of 3 study groups in a 2:2:1 ratio (self-selected vs assigned vs control). Participants in the self-selected group were presented with 3 target options: 10%, 20%, and 30%, and they were asked to self-select a target to reduce their mobile screen time from their baseline average mobile screen time. Participants in the assigned group were given a target to reduce their mobile screen time from their baseline average mobile screen time. The assigned target was set as the average of the targets selected by participants in the self-selected group. During the intervention period, participants in the self-selected and assigned group were eligible to receive a monetary incentive of INR (Indian Rupee) 50 (US $0.61) per day for successfully attaining their target. Participants in the control group neither received nor selected a target for reducing their mobile screen time and did not receive any monetary incentives during the intervention period. All participants received information regarding the advantages of reducing mobile screen time. As an incentive, all participants would receive INR 500 (US $6.06) upon completion of the study and a chance to win 1 of 2 lotteries valued at INR 5000 (US $60.55) for consistently sharing their mobile screen time data.

**Results:**

Currently, the study intervention is being rolled out. Enrollment occurred between August 21, 2023, and September 2, 2023; data collection concluded in November 2023. We expect that results will be available by early 2024.

**Conclusions:**

The monetary incentives and self-selected versus assigned targets might be effective interventions in reducing mobile screen time among working professionals and students.

**Trial Registration:**

AsPredicted 142497; https://aspredicted.org/hr3nn.pdf

**International Registered Report Identifier (IRRID):**

DERR1-10.2196/53756

## Introduction

Smartphones have become ubiquitous in people’s lives. The global average for daily screen time is 6 hours and 37 minutes [1]. In 2021, India had a mobile subscriber count of 1.2 billion, and around 750 million of them were using smartphones [2]. The average screen time for Indians has increased to 7.3 hours [3]. As of 2019, the leading mental disorders are depression and anxiety, which affect 280 million and 301 million people worldwide, respectively [4]. Estimates suggest that over 197 million Indians, approximately 15% of our population, experience mental disorders. Of these, approximately 85 million experience depression- and anxiety-related disorders [5]. Existing research links excessive screen time to lower subjective well-being, depression, anxiety, sleep disorders, and poor physical health [6-13].

Numerous correlational studies have explored the relationship between digital media use and well-being, with many of them indicating that screen time and mobile consumption are negatively linked to subjective well-being [6-10,14]. For instance, Przybylski and Weinstein [15] explored the effect of digital media use on the well-being of adolescents, providing support for the Goldilocks hypothesis—individuals with moderate use have higher well-being than those with lower and higher use.

On the other hand, Twenge and Campbell [14] showed that although light users of digital media had higher well-being than people who abstain, moderate and heavy users had lower well-being than light users. Twenge and Martin [7] demonstrated that the associations between greater digital media use and psychological well-being are greater for female than male individuals. In their correlational study, Twenge and Campbell [14] found that individuals with moderate and heavy digital media use (smartphones, social media, and others) had lower well-being than those with lighter media use, especially individuals with greater than 5 hours of digital media use per day. Orben and Przybylski [16] used specification curve analysis to understand if there is any correlational relationship between digital media use and well-being among adolescents. They found a negative but small association, explaining at most 0.4% of the variation in well-being [16].

In addition, existing research has also shown that temporary monetary incentives and targets can be used to inculcate behavioral change in domains such as gym attendance [17], smoking, work behavior [18], alcohol abuse [19], and weight loss [20]. Research studies suggest that temporary monetary incentives and specific targets can effectively reduce social media use and allow people to limit their screen time [21].

However, the current body of research does not provide evidence regarding whether self-selected targets or externally imposed targets are more effective in promoting and maintaining behavioral change, especially in the context of smartphone use, thereby improving mental health. Reducing smartphone screen time is essential for maintaining a healthy and productive lifestyle.

In this study, we aim to nudge people to reduce their screen time by providing temporary monetary incentives and targets. Target is defined as the minimum percentage reduction in daily mobile screen time from the daily average mobile screen time during the baseline period.

The objectives of the study are as follows:

1. To estimate the impact of monetary incentives and targets on reducing mobile screen time during the treatment and posttreatment period.
2. To evaluate the efficacy of self-selected versus assigned targets in reducing mobile screen time.
3. To estimate the impact of screen time reduction on mental health. Mental health will be assessed using measures such as Generalized Anxiety Disorder–7 (GAD-7) for anxiety, Patient Health Questionnaire–9 (PHQ-9) for depression, daily mood survey for mood, and well-being scores for general well-being.

## Methods

### Study Design and Setting

To achieve the study objectives, we are conducting a pragmatic, 3-arm, parallel-group, randomized controlled trial involving students and employees of the Indian School of Business (ISB). The ISB operates across 2 campuses located in the states of Punjab and Telangana, India. The randomized controlled trial spans a 12-week duration, comprising 2 weeks of baseline, 1 week for randomization, 5 weeks of intervention, and 4 weeks of the postintervention period.

### Participants and Eligibility Criteria

Current employees and students at both ISB campuses who had Android-operated mobile phones and were available throughout the 12-week study period were eligible to participate in the study. The study excluded employees affiliated with the implementing department, Max Institute of Healthcare Management, ISB, as they previously participated in the study’s pilot.

### Recruitment

The study was conducted entirely through digital communication, avoiding the need for in-person interaction between the researchers and the participants. We initiated the study by sending an email through the Head of Student Affairs and Human Resources to both employees and students of ISB to their official email addresses. The email included a link to a form that allowed participants to express their interest in the study. This email was sent 3 times. Recruitment took place for 2 weeks, the preregistration period, to gather expressions of interest. Potential participants who expressed interest were subsequently provided with study details and consent forms. Three email reminders were sent to the potential participants to provide consent. The participants who provided consent were asked to complete the baseline survey and provide their mobile screen time data during the baseline period.

### Randomization and Blinding

**Figure 1:**
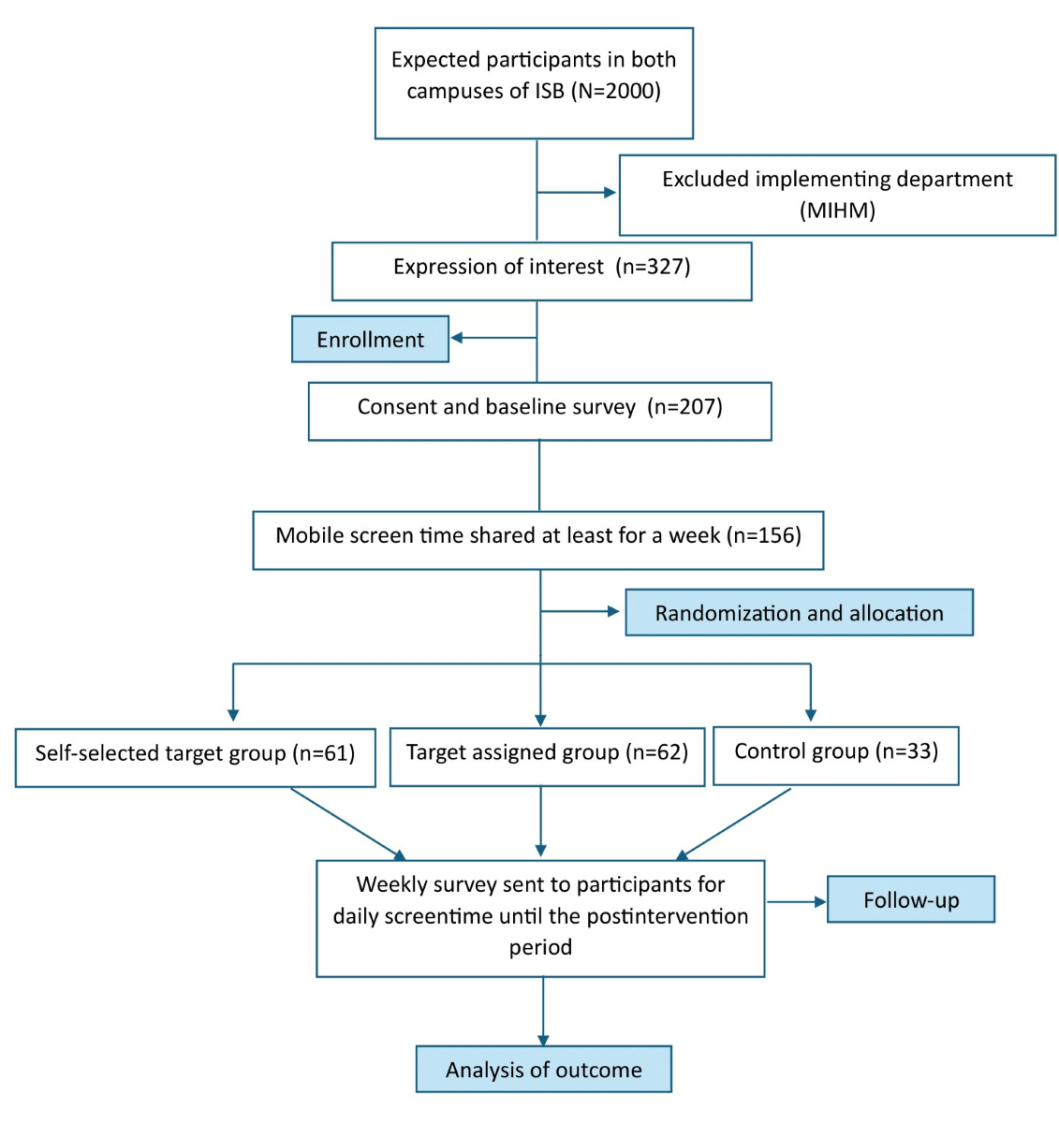
The CONSORT (Consolidated Standards of Reporting Trials) flow diagram for the study.

After obtaining the baseline mobile screen time data, participants were randomly assigned to 1 of 3 groups in a 1:2:2 (control vs self-selected vs assigned) ratio. This assignment was carried out using a stratified randomized permuted block design with a block size of 5 using the *ralloc* command on Stata 17 software (StataCrop LLC). The allocation was stratified based on 2 variables: status in the institute (employee or student) and the quartile of baseline average mobile screen time. Following randomization, the research team became aware of the allocation groups. Blinding at the participant level was not possible due to the nature of the intervention. Participants were informed about their group before the start of the intervention period. They were not told about other groups or the groups of other participants. The CONSORT (Consolidated Standards of Reporting Trials) flow diagram for the study is provided in Figure 1.

### Sample Size

The target sample was 300 participants, assuming a conservative effect size (*d*) of 0.12 for the difference between self-selected and assigned conditions and 0.15 for the difference between the intervention group and control conditions, with 80% power and an α of .05. The effect size was estimated based on past studies in this domain [22,23].

### Interventions

At the end of the baseline period, the participants were randomized into 3 groups: the control group and 2 intervention groups.

#### Intervention-1 (Self-Selected Group)

In this group, participants were asked to select a target to reduce their mobile screen time from their baseline average mobile screen time. They were presented with 3 target options (10%, 20%, and 30%) and were asked to select one of the targets. The target options were chosen such that none of the targets is unreasonably high, ensuring that there are at least some participants who chose the target. Additionally, we want to ensure that the targets are sufficiently different so that participants are not indifferent between them. During the intervention period, participants in this group were eligible to receive a monetary incentive of INR (Indian Rupee) 50 (US $0.61) per day for successfully achieving the target they selected.

#### Intervention-2 (Assigned Group)

In this group, participants were assigned a target to reduce their mobile screen time from their baseline average. This target was determined after the participants in the self-selected group chose their respective targets. The assigned target was determined as the average of the targets selected by participants in the self-selected group. This was done such that both groups have the same average screen time reduction target, and thereby we can compare the average screen time reduction between the groups. This makes the intervention arms on average only differ in whether they chose or were assigned a screen time reduction target. During the intervention period, participants in this group were eligible to receive a monetary incentive of INR 50 (US $0.61) per day for successfully achieving the assigned target.

#### Control Group

Control group participants were neither assigned nor asked to select a target for reducing their mobile screen. They were provided with standard information regarding the advantages of reducing their mobile screen time and received reminders to do so. They will not receive any monetary incentives for reducing their mobile screen time.

All participants, including the control group, received identical information regarding the advantages of reducing mobile screen time. All participants were informed that they would receive a fixed amount of INR 500 (US $6.06) upon completing the study. Additionally, participants in the study who report their mobile screen time every week will have the opportunity to win 1 of 2 lotteries, worth INR 5000 (US $60.55) each.

### Study Procedures and Stages

**Figure 2:**
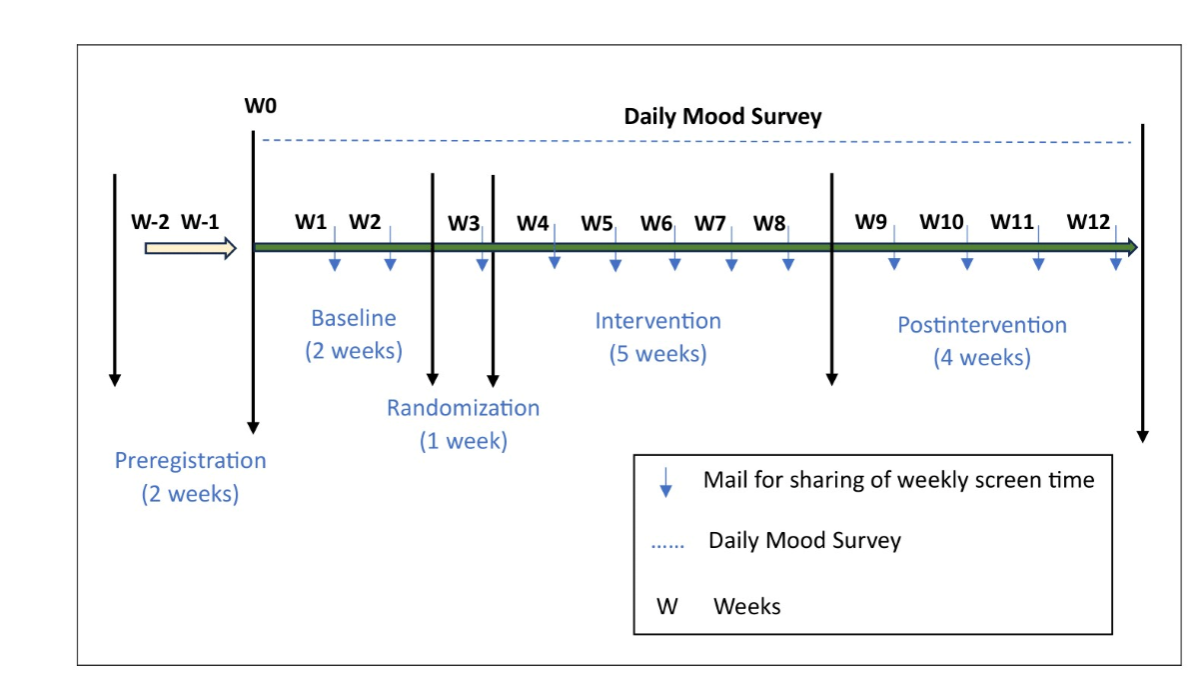
An overview of the study.

The study has 5 stages: preregistration, baseline, group allocation, intervention, and postintervention, as presented in Figure 2.

#### Preregistration

Over 2 weeks, we conducted preregistration to gather expressions of interest. A total of 327 individuals expressed interest in the study. The individuals who displayed interest in the study were requested to complete the baseline survey, which included information regarding demographics, daily mobile phone time, past attempts to reduce screen time, willingness to reduce screen time, and GAD-7 and PHQ-9 for assessing anxiety and depression (a list of collected data is summarized in Table 1). Participants were also asked to report their daily mobile screen time and upload screenshots of the digital well-being app for the week before preregistration.

**Figure 3:**
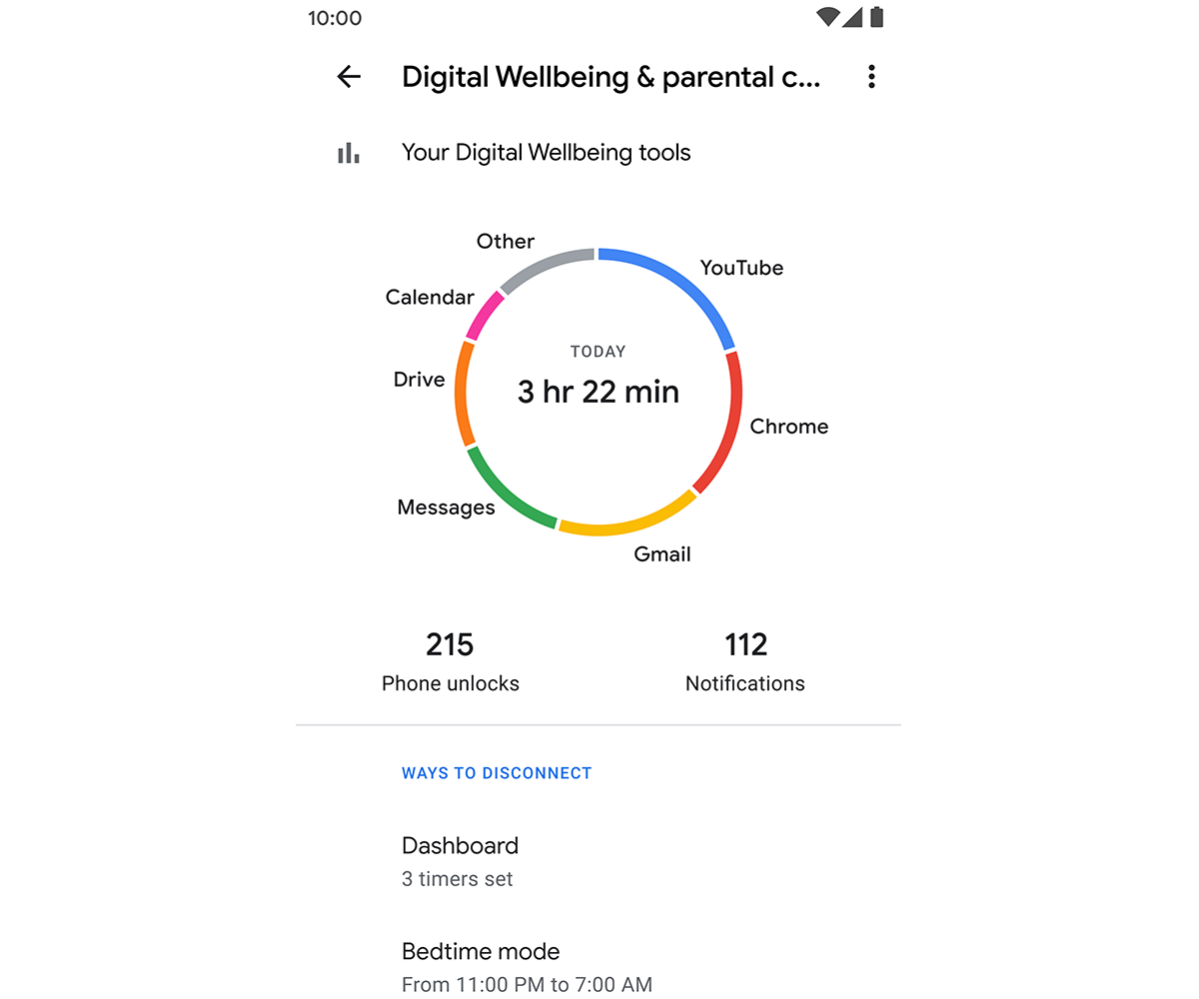
A screenshot of the digital well-being app.

The digital well-being tools offer a daily overview of a person’s phone use: the number of times the user checks their phone and the screen time. The screen time measures the total and app-wise time of the phone use. Participants share their mobile screen time through a weekly survey asking them to fill in the total time in hours and minutes of screen time for each day as displayed in the digital well-being app. As proof, the participants were asked to upload a screenshot of the digital well-being app for that week. A screenshot of the digital well-being app is shown in Figure 3. The study team checks whether the screen time in the survey matched with the screenshot. The study team provided step-by-step visual instructions for the process of uploading the screenshot from the digital well-being app. Of the 327 individuals interested in the study, 207 provided consent and completed the baseline survey.

**Table 1:** Data collection at various stages of the study.

Data category	Expression of interest	Baseline^a,b^	End of intervention^a,b^	End of postintervention^a,b^
Basic information and contact details	✓	✓	✓	✓
Demographic		✓		
Information on willingness, difficulty, and magnitude in reducing mobile screentime		✓		
Information about mobile use		✓		✓
Phone addiction scale	✓	✓		✓
Generalized Anxiety Disorder–7		✓	✓	✓
Patient Health Questionnaire–9		✓	✓	✓

^a^Daily mobile screen time use: participants share their daily screen time and upload screenshots of their screen time by using the digital well-being feature on their Android mobile phones through self-administered weekly surveys.

^b^Daily mood survey: mood data are collected daily through WhatsApp messages, using a 1-10 scale that is sent at varied times of the day.

#### Baseline (Mobile Screen Time)

The 2-week period following the preregistration was the baseline period. Every Sunday during this period, we emailed participants who had completed the baseline survey. They were instructed to provide their daily mobile screen time for the preceding week and upload a screenshot of the digital well-being app on their phones. Two reminder emails were sent to those who did not respond. Additionally, participants received a daily mood survey via WhatsApp (Meta) every day. Throughout the 2-week baseline period, 156 participants shared their mobile screen time for at least 1 week, while those who did not respond were excluded from the study.

#### Group Allocation

The 1-week period after the baseline was the group allocation period. During this period, the 156 participants who shared their mobile screen data were randomly assigned to 1 of 3 study groups. Initially, participants in the self-selected group were given information regarding their baseline average daily mobile screen time and asked to choose a target for reducing their mobile screen time based on this average. After the participants in the self-selected group had made their specific target choices, an email was sent to participants in the assigned group informing them about their given target.

All participants, including the control groups, received an email about the benefits of reducing mobile screen time and their baseline average daily mobile screen time.

#### Intervention Period

The period of 5 weeks following the group allocation is the intervention period. During this, the participants will receive an email every Sunday asking them to report their mobile screen time for the preceding week and provide a screenshot. Two reminder emails will be sent to those who do not respond. Additionally, participants will continue to receive a daily mood survey through WhatsApp. Every Wednesday, we will send out reminders to all groups via WhatsApp with information about the benefits of reducing mobile screen time and details about 2 lotteries. Participants in both intervention groups will also receive email reminders for their chosen and assigned targets. Participants in the intervention groups will receive a daily incentive of INR 50 (US $0.61) for successfully meeting their daily target. These incentives will be transferred to the participants via the Unified Payments Interface after the intervention period. At the end of the intervention, an end-of-intervention survey will be administered to all participants.

#### Postintervention Period

The 4-week period following the intervention period is the postintervention period. During this, the participants will continue to receive an email requesting them to report their mobile screen time for the previous week and provide a screenshot. Two reminder emails will be sent to the participants who do not respond. Participants will also continue to receive a daily mood survey through WhatsApp. The participants will not receive monetary incentives for achieving their targets during this phase. After the postintervention period, an end-of-postintervention survey will be conducted.

### Data Collection and Management

The following data are collected during various stages of the study (Table 1). Detailed survey instruments are presented in Multimedia Appendix 1.

We collect data through a web-based survey link shared over an email. All survey instruments are created using Qualtrics (Qualtrics International Inc), a secure web-based survey and database administration application. Data privacy is maintained with password protection for access to the Qualtrics platform. Only members of the research team have access to the database. We use the user-friendly WhatsApp platform for the daily mood survey to gather mood ratings on a scale. Upon concluding the study, the mood survey data will be extracted from WhatsApp and integrated with other data for subsequent analysis.

A pilot study was conducted with the implementing department (Max Institute of Healthcare Management) employees to evaluate and refine the study procedures and the survey tools.

### Statistical Analysis

We will estimate the effects of mobile screen time, target achievement rate, GAD-7, PHQ-9, and daily mood scores by comparing variables of interest between the intervention and baseline and between the postintervention and baseline periods. To perform this analysis, we will use Stata 17 and R (version 4.2.2; Free Open-Source Software). The unadjusted effects will be calculated as differences in the means and conducting a 2-tailed Student *t* test for significance. For adjusted effects, we will use simple ordinary least square regression adjusting for participant-level covariates: age, gender, and employee or student. We assume errors to be correlated within each participant but uncorrelated across participants. A separate equation will be used for each dependent variable. The regression equations are provided in Multimedia Appendix 1. For mobile screen time, we will estimate a difference-in-difference ordinary least square regression. To account for the impact of the intervention and the postintervention period, we will incorporate dummy variables for the period. Specifically, assessing the effect on mobile screen time and target achievement rate postintervention will provide valuable insights into habit formation. Multilevel or hierarchical linear modeling will also be used to allow differential relationships between mood and screen time at the individual level.

### Ethical Considerations

Ethical approval for this study was granted by the institutional review board of the ISB, Hyderabad, India (reference ISB-IRB2023-18; dated July 26, 2023).

## Results

We started the recruitment process on August 21, 2023. We randomized 156 participants, and at the original submission of the protocol, we completed 3 weeks of the intervention period. The results are expected to be published in early 2024. The study results will be published in academic journals. These published findings have the potential to guide policy makers in establishing guidelines for monitoring and limiting excessive mobile screen exposure among both working professionals and students.

## Discussion

### Principal Findings

The study focuses on the impact of monetary incentives and self-selected versus assigned targets on reducing mobile screen time and thereby improving mental health and well-being during the intervention and postintervention periods.

Previous studies mainly focused on providing correlational evidence. For example, Mosquera et al [24] found that deactivating Facebook for 1 week made people less depressed and led them to engage in healthier activities. Allcott et al [25] found that deactivating Facebook for 4 weeks before the 2018 US election caused small but significant improvements in well-being, self-reported happiness, life satisfaction, depression, and anxiety. However, they did not find an effect on other well-being measures. In a follow-up paper, Allcott et al [21] monetarily incentivized people to reduce social media (Facebook, Instagram, Twitter, Snapchat, web browsers, and YouTube). The group that was monetarily incentivized over a period reduced their screen time by 56 minutes during the treatment period of 3 weeks. However, they found statistically insignificant changes in measures of happiness, life satisfaction, anxiety, and depression [21].

Compared with existing studies that have incentivized reduction over a period, this study offers everyday incentives and targets for people to reduce their overall smartphone use. While there is only 1 study that has used daily incentives, the focus of the study is on rational addiction and does not test the effects on anxiety and depression [22]. In addition, none of the studies have examined self-chosen targets versus given targets on the reduction, target achievement, and impact on well-being. Moreover. the existing studies do not study the effect of reduction on PHQ-9, GAD-7, and daily moods. Although Alcott et al [25] measured daily happiness, they did not have daily use data to understand the impact of daily use on happiness [25].

In this study, we expect the screentime reduction target to vary at the individual level. There might be cases when the average target is higher than the individual reduction that some individuals will prefer to achieve, while for some others there might be cases when the target is less than the individual reduction they will aim to achieve. In the former case, the participants may not be able to achieve the average target, while in the latter case, they will achieve it. Other psychological factors, such as cognitive dissonance, support the idea that the self-selected condition is likely to outperform the assigned target condition. This is because participants are motivated to follow through on their personally chosen targets. So overall the reduction in exogenous (assigned group) condition could be lower due to this reason as well.

Through rigorous research design and a comprehensive examination of various factors, the results from the study make a substantial contribution to enhancing the comprehension of how self-selected versus assigned targets may potentially reduce mobile screen use and thereby improve mental health.

### Strengths

The primary strength of the study is its intervention, which involves the use of monetary incentives and self-selected versus assigned to reduce mobile screen time and assess improvements in mental health through measures such as the GAD-7, PHQ-9, daily mood scores, and well-being scores. We will have daily mobile screen use data and therefore assess the impact of daily targets and incentives on daily screen use. Another strength advantage is that the study is conducted using digital communication, which removes the requirement for physical contact with participants.

### Limitations

One of the potential limitations of the study is that the analyses in the study may lack sufficient power to detect the anticipated effects due to low enrollment. Another limitation is requesting the weekly submission of screen time through a mobile app, which might increase overall mobile screen time. However, this task only demands 4-5 minutes of weekly effort and will not impact the results, as the same procedure was also used at the baseline. The daily mood score was collected through a messaging app, WhatsApp, which is another limitation of the study.

### Conclusions

The results of the study might add a significant layer of depth to the existing knowledge, guiding both researchers and policy practitioners toward more about monetary incentives, as well as self-selected versus assigned targets that might be effective interventions in changing health behavior including mobile screen use and thereby improving mental health for working professional and students.

## Appendix

Multimedia Appendix 1 Surveys, weekly reminders and emails, and statistical analysis.

## Abbreviations

CONSORT: Consolidated Standards of Reporting Trials
GAD-7: Generalized Anxiety Disorder–7
ISB: Indian School of Business
INR: Indian Rupee
PHQ-9: Patient Health Questionnaire–9

## Data Availability

The data sets generated or analyzed during this study are available from the corresponding author on reasonable request. The data collected for this study will be stored in the central repository of the Indian Schools of Business.
