# Supplementary material for: Self-Selected Versus Assigned Target to Reduce Smartphone Use and Improve Mental Health: Protocol for a Randomized Controlled Trial
Source: JMIR Res Protoc. 2024 May 6;13:e53756. doi: 10.2196/53756 (PMC11106705; doi:10.2196/53756)
Supplement: Multimedia Appendix 1 [file resprot_v13i1e53756_app1.docx]

**Self-selected vs. Assigned target to reduce smartphone usage and improve mental health: Protocol for a Randomized Controlled Trial**

**Supplementary materials:**

Contents

[**1.** **Surveys** 1](#_Toc164828249)

[**1.1.** Baseline Survey 1](#_Toc164828250)

[**1.2.** End of intervention survey 13](#_Toc164828251)

[**1.3.** End of post-intervention survey 21](#_Toc164828252)

[**2.** **Weekly What’s App reminders:** 29](#_Toc164828253)

[**3.** **Weekly mail for sharing screen time:** 29](#_Toc164828254)

[**3.1.** **For Intervention groups** 30](#_Toc164828255)

[**3.2.** **For control group** 30](#_Toc164828256)

[**4.** **Statistical analysis: Regression equations** 31](#_Toc164828257)

1. **Surveys**
   1. Baseline Survey

Stay Well
Participant Consent Form

Hello.
You are invited to participate in a research study “Stay Well” conducted by the Max Institute of Healthcare Management, Indian School of Business.
 1. Details of the study:  
 The study will be conducted over 12 weeks. Participation in this study is voluntary. All the study communication will happen virtually so your physical presence is not necessary.                   
       
 2. What you have to do:
 During the study, you will be:
 2.1 Asked to activate the digital well-being feature in your Android phones (Step-by-step instructions are attached along with this email).
 2.2 Required to upload a screenshot of your mobile usage and report it at the end of every week. Note: We are only interested in your total usage and not in any specific apps that you are using.
 2.3 Asked to complete three surveys. It will take approximately 10 minutes to fill out each survey.
 2.4 Asked to rate your daily mood via WhatsApp every day, which will require approximately 10 seconds to respond.     
  
  3. Benefits and Risks:
 3.1 Participating in this study will help you manage your mobile screen time better and potentially improve your mental health.
 3.2 You will earn at least Rs 500 for your participation, and those who upload all screenshots will get a chance to participate in a lottery that pays 2 prizes of Rs 5000.   
 3.3 There are no known risks associated with your participation in this research beyond those of everyday life.
 3.4 There will be no consequences for not completing the study. You may drop out of the study at any time and for any reason.   
                                
 4. Data Privacy:
 All screenshots and responses will be anonymized and kept confidential. Any information we include in our research will not identify you as the respondent.

 If there is any question about the study, you may contact the Principal Investigator: Ashish Sachdeva at phone 01724591831 or email ashish_sacheva@isb.edu at the Indian School of Business, Sector 81, Mohali, Punjab – 140306, India.

 For questions about your rights as a research participant, you may contact the Chair of the Institutional Review Board (IRB) at ISB: Professor Ashwini Chhatre at 040-2318-7134 or email ashwini_chhatre@isb.edu at the Indian School of Business, Gachibowli, Hyderabad – 500111, India.

 1. Do you consent to be a part of the study?

- Yes (4)
- No (5)

2. Please enter your ISB email ID (for example: john_doe@isb.edu).

________________________________________________________________

3. UPI For payment purpose: Please enter your valid UPI ID (In case you don't have a UPI ID mention NA. We will contact you separately).

________________________________________________________________

4. What is your age (in years)?

________________________________________________________________

5. Please enter your gender.

- Male (1)
- Female (2)
- Non-binary (3)
- Prefer not to say (4)

6. Are you an ISB employee or student?

- Employee (1)
- Student (2)

7. Please enter your City and State.

- City (1) __________________________________________________
- State (2) __________________________________________________

8. How many total months have you worked full-time in a professional organizations? In case of no prior work experience please enter 0.

________________________________________________________________

9. On a typical day, how long do you use your mobile phone(s) on average? (average_usage)
 (Please refer to the attached step-by-step guide to access the digital well-being feature of your Android phone and provide an estimate for daily average in terms of hours and minutes).

- Hours (1) __________________________________________________
- Minutes (2) __________________________________________________

10. How many mobile phones do you currently have?

- 1 (1)
- 2 (2)
- More than 2 (3)

11.What proportion of your overall screen time do you spend on each of the following devices?  The sum total should be 100%.

Mobile Phone : _______ (1)

Tablet : _______ (2)

Laptop : _______ (3)

Others : _______ (4)

Total : ________

12. What proportion of your mobile phone screen time do you spend on the following categories?
 Please note that the proportions should add up to 100%.

Social Networking : _______ (1)

Entertainment : _______ (2)

Gaming : _______ (3)

Productive activities : _______ (4)

Others : _______ (5)

Total : ________

13. Have you ever tried to reduce your mobile phone usage?

- Yes (1)
- No (2)

14. How difficult was it to reduce your mobile phone usage?

|  | 1 | 2 | 3 | 4 | 5 | 6 | 7 |
| --- | --- | --- | --- | --- | --- | --- | --- |

| 1- Not at all difficult to 7- Most difficult. () | 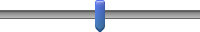 |
| --- | --- |

15. How willing are you to reduce your mobile phone usage?

|  | 1 | 2 | 3 | 4 | 5 | 6 | 7 |
| --- | --- | --- | --- | --- | --- | --- | --- |

| 1- least willing to 7- most willing () | 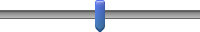 |
| --- | --- |

16. If given a chance, how much percentage of your current daily average mobile screen time you will aim to reduce? (If you choose 0 %, you don't aim to reduce your mobile screen time. If you choose 100%, you want to abstain completely from using your mobile phone).

|  | 0 | 10 | 20 | 30 | 40 | 50 | 60 | 70 | 80 | 90 | 100 |
| --- | --- | --- | --- | --- | --- | --- | --- | --- | --- | --- | --- |

| % () | 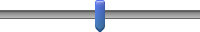 |
| --- | --- |

17. Do you set a goal or target to motivate yourself to change any behavior?

|  | 1 | 2 | 3 | 4 | 5 | 6 | 7 |
| --- | --- | --- | --- | --- | --- | --- | --- |

| (1 - Not at all, 7-Very often) () | 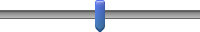 |
| --- | --- |

18. How difficult goal or target do you set to motivate yourself?

|  | 1 | 2 | 3 | 4 | 5 | 6 | 7 |
| --- | --- | --- | --- | --- | --- | --- | --- |

| (1- Easily achievable goals, 7- Very difficult goals) () | 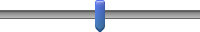 |
| --- | --- |

19. Do you have self-control problems to meet the set goals?

|  | 1 | 2 | 3 | 4 | 5 | 6 | 7 |
| --- | --- | --- | --- | --- | --- | --- | --- |

| 1- Not at all to 7- Very much () | 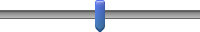 |
| --- | --- |

20. In the past three weeks, how often have you

|  | Never (1) | Rarely (2) | Sometimes (3) | Often (4) | Always (5) |
| --- | --- | --- | --- | --- | --- |
| Felt anxious when you don't have your phone (35) |  |  |  |  |  |
| Preferred to use phone over interacting with friends or family (36) |  |  |  |  |  |
| Felt worried about missing things online when you are not checking your phone (37) |  |  |  |  |  |
| Lost your sleep due to using phone at night (38) |  |  |  |  |  |
| Felt your performance in school or at work suffers because of the amount of time you use your phone (39) |  |  |  |  |  |
| Found it difficult to switch off or put down your phone? (40) |  |  |  |  |  |
| Using your phone longer than intended (42) |  |  |  |  |  |
| Checked your phone immediately after waking up (43) |  |  |  |  |  |

21. Over the last 2 weeks, how often have you been bothered by any of the following problems? (0 - Not at all, 1 – Several days, 2 – More than half the days, 3 – Nearly every day)

|  | Not at all | Several days | More than half the days | Nearly every day |
| --- | --- | --- | --- | --- |

|  | 0 | 1 | 2 | 3 |
| --- | --- | --- | --- | --- |

| Little interest or pleasure in doing things () | 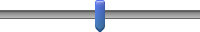 |
| --- | --- |
| Feeling down, depressed, or hopeless () | 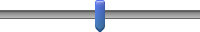 |
| Trouble falling or staying asleep, or sleeping too much () | 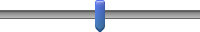 |
| Feeling tired or having little energy () | 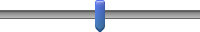 |
| Poor appetite or overeating () | 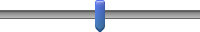 |
| Feeling bad about yourself – or that you are a failure or have let yourself or your family down () | 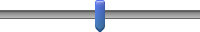 |
| Trouble concentrating on things, such as reading the newspaper or watching television () | 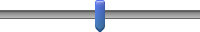 |
| Moving or speaking so slowly that other people could have noticed. Or the opposite – being so restless that you have been moving around a lot more than usual () | 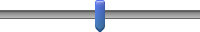 |
| Thoughts that you would be better off dead, or of hurting yourself in some way () | 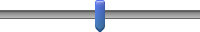 |

22. Over the last 2 weeks, how often have you been bothered by any of the following problems? (0 - Not at all, 1 – Several days, 2 – More than half the days, 3 – Nearly every day)

|  | Not at all | Several days | More than half the days | Nearly every day |
| --- | --- | --- | --- | --- |

|  | 0 | 1 | 2 | 3 |
| --- | --- | --- | --- | --- |

| Feeling nervous, anxious, or on edge () | 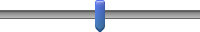 |
| --- | --- |
| Not being able to stop or control worrying () | 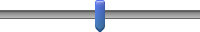 |
| Worrying too much about different things () | 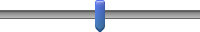 |
| Trouble relaxing () | 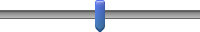 |
| Being so restless that it is hard to sit still () | 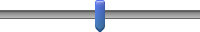 |
| Becoming easily annoyed or irritable () | 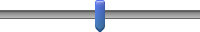 |
| Feeling afraid, as if something awful might happen () | 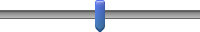 |

23. On a scale of 1-10 (1- Being not satisfied at all to 10- Being extremely satisfied), how satisfied are you with your life?

|  | Not satisfied at all | Extremely satisfied |
| --- | --- | --- |

|  | 1 | 2 | 3 | 4 | 5 | 6 | 7 | 8 | 9 | 10 |
| --- | --- | --- | --- | --- | --- | --- | --- | --- | --- | --- |

| 1- Being not satisfied at all to 10- Being extremely satisfied () | 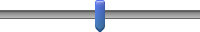 |
| --- | --- |

24. Would you prefer to receive Rs 200 today or Rs 250 in one month?

- Rs 200 today (1)
- Rs 250 in one month (2)

25. Would you prefer to receive Rs 200 in 6 months of Rs 250 in 7 months?

- Rs 200 in 6 months (1)
- Rs 250 in 7 months (2)

26. Please upload the screenshot of your Digital Wellbeing app here.
<<INSERT IMAGE>>
Make sure the average screen time (or total screen time) over the past week is visible as the bar graph.
 The screenshot above is from an Android device.
 
This is mainly to confirm if your Digital Wellbeing app is working well. Please do not deactivate the app during the course of the experiment.
 
Note: Please refer to the step-by-step guide to use the Digital Wellbeing feature attached to this email.

27. If you have any comments or remarks, please use the text box below. 

We will be in touch with you via email in a few days to send you the next instructions. Please click "Next" to submit your responses.

________________________________________________________________

________________________________________________________________

________________________________________________________________

________________________________________________________________

________________________________________________________________

- 1. End of intervention survey

Q1 Consent Form
Hello. 
This is a survey for the study titled “Stay Well”.
 This survey will take 5 minutes to complete. 
 There are no known risks associated with your participation in this research beyond those of everyday life. All responses will be kept confidential, and deidentified data will be used for analysis. Participation in this study is voluntary. There will be no consequences for not completing the study. You may drop out of the study at any time and for any reason.
 If there is anything about the study or your participation that is unclear or that you do not understand, if you have questions or wish to report research–related problem, you may contact the Principal Investigator: Ashish Sachdeva at phone 01724591831 or email ashish_sacheva@isb.edu at the Indian School of Business, Sector 81, Mohali, Punjab – 140306, India.
 For questions about your rights as a research participant, you may contact the Chair of the Institutional Review Board (IRB) at ISB: Professor Ashwini Chhatre at 040-2318-7134 or email ashwini_chhatre@isb.edu at the Indian School of Business, Gachibowli, Hyderabad – 500111, India.

 Do you consent to participate in this survey?

- Yes (1)
- No (2)

|  |
| --- |

Q2 Please enter your ISB email ID.

________________________________________________________________

Q3 From September 24 to October 28, 2023, you had a target to reduce your mobile screen time, and you were rewarded with Rs 50 for each day you achieved this target.
 For the next four weeks, there won't be any monetary rewards for achieving the target, but we strongly encourage you to continue reducing your mobile screen time.

 Please confirm your understanding that you will not receive any monetary rewards for reducing your mobile screen time going forward. (Only asked to participants in intervention groups)

- Yes, I acknowledge (7)

Q4 What will be your average daily mobile screen time for the next four weeks (28 days)?

- Hours (1) __________________________________________________
- Minutes (2) __________________________________________________

Q5 Did you try to reduce your mobile screen time during the last five weeks?

- Yes (1)
- No (2)

Q6 How difficult was it to reduce your mobile screen time and meet your target in the last five weeks? (Asked only to participants in intervention groups)

  (1- least difficult to 7- most difficult)

|  | Least difficult | Most difficult |
| --- | --- | --- |

|  | 1 | 2 | 3 | 4 | 5 | 6 | 7 |
| --- | --- | --- | --- | --- | --- | --- | --- |

| 1- least difficult to 7- most difficult () | 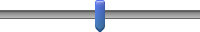 |
| --- | --- |

Q7 During the last five weeks, what was your main strategy to reduce your mobile screen time?

- Use features of a Digital well-being app (e.g. tracking screen time, etc.) (1)
- Reduce notifications from my phone (e.g. turning off notifications/sounds). (2)
- Delete some apps. (3)
- Reduce my social networking (e.g. Instagram, Tiktok, Facebook) (4)
- Use my laptop/tablet more (5)
- Others (please describe in the text box below) (6) __________________________________________________

Q8 Did you develop a new habit or routine to replace the time you saved by decreasing your screen time? (1- Not at all to 7- Very Much)

|  | Not at all | Very Much |
| --- | --- | --- |

|  | 1 | 2 | 3 | 4 | 5 | 6 | 7 |
| --- | --- | --- | --- | --- | --- | --- | --- |

| (1- Not at all to 7- Very Much) () | 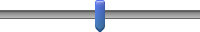 |
| --- | --- |

Q9 If given a chance again, what percentage of your baseline mobile screen time will you aim to reduce? (If you choose 0 %, you don't aim to reduce your mobile screen time. If you choose 100%, you want to abstain completely from using your mobile phone) (Asked only to Self-selected target group)

|  | 0 | 10 | 20 | 30 | 40 | 50 | 60 | 70 | 80 | 90 | 100 |
| --- | --- | --- | --- | --- | --- | --- | --- | --- | --- | --- | --- |

| % () | 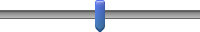 |
| --- | --- |

10. In the past three weeks, how often have you

|  | Never (1) | Rarely (2) | Sometimes (3) | Often (4) | Always (5) |
| --- | --- | --- | --- | --- | --- |
| Felt anxious when you don't have your phone (35) |  |  |  |  |  |
| Preferred to use phone over interacting with friends or family (36) |  |  |  |  |  |
| Felt worried about missing things online when you are not checking your phone (37) |  |  |  |  |  |
| Lost your sleep due to using phone at night (38) |  |  |  |  |  |
| Felt your performance in school or at work suffers because of the amount of time you use your phone (39) |  |  |  |  |  |
| Found it difficult to switch off or put down your phone? (40) |  |  |  |  |  |
| Using your phone longer than intended (42) |  |  |  |  |  |
| Checked your phone immediately after waking up (43) |  |  |  |  |  |

11. Over the last 2 weeks, how often have you been bothered by any of the following problems? (0 - Not at all, 1 – Several days, 2 – More than half the days, 3 – Nearly every day)

|  | Not at all | Several days | More than half the days | Nearly every day |
| --- | --- | --- | --- | --- |

|  | 0 | 1 | 2 | 3 |
| --- | --- | --- | --- | --- |

| Little interest or pleasure in doing things () | 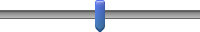 |
| --- | --- |
| Feeling down, depressed, or hopeless () | 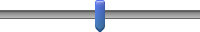 |
| Trouble falling or staying asleep, or sleeping too much () | 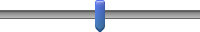 |
| Feeling tired or having little energy () | 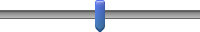 |
| Poor appetite or overeating () | 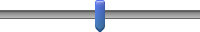 |
| Feeling bad about yourself – or that you are a failure or have let yourself or your family down () | 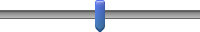 |
| Trouble concentrating on things, such as reading the newspaper or watching television () | 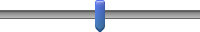 |
| Moving or speaking so slowly that other people could have noticed. Or the opposite – being so restless that you have been moving around a lot more than usual () | 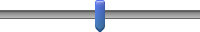 |
| Thoughts that you would be better off dead, or of hurting yourself in some way () | 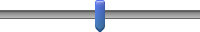 |

12. Over the last 2 weeks, how often have you been bothered by any of the following problems? (0 - Not at all, 1 – Several days, 2 – More than half the days, 3 – Nearly every day)

|  | Not at all | Several days | More than half the days | Nearly every day |
| --- | --- | --- | --- | --- |

|  | 0 | 1 | 2 | 3 |
| --- | --- | --- | --- | --- |

| Feeling nervous, anxious, or on edge () | 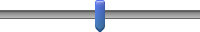 |
| --- | --- |
| Not being able to stop or control worrying () | 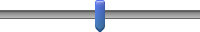 |
| Worrying too much about different things () | 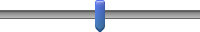 |
| Trouble relaxing () | 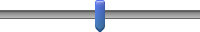 |
| Being so restless that it is hard to sit still () | 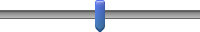 |
| Becoming easily annoyed or irritable () | 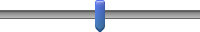 |
| Feeling afraid, as if something awful might happen () | 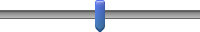 |

13. Satisfaction On a scale of 1-10 (1- Being not satisfied at all to 10- Being extremely satisfied), how satisfied are you with your life?

|  | Not satisfied at all | Extremely satisfied |
| --- | --- | --- |

|  | 1 | 2 | 3 | 4 | 5 | 6 | 7 | 8 | 9 | 10 |
| --- | --- | --- | --- | --- | --- | --- | --- | --- | --- | --- |

| 1- Being not satisfied at all to 10- Being extremely satisfied () | 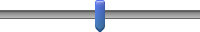 |
| --- | --- |

14. If you have any comments or remarks, please use the text box below.

________________________________________________________________

- 1. End of post-intervention survey

Q1 Please enter your ISB email ID.

________________________________________________________________

Q2 Consent Form
Hello. 
This is a survey for the study titled “Stay Well”.
 This survey will take 5 minutes to complete. 
 There are no known risks associated with your participation in this research beyond those of everyday life. All responses will be kept confidential, and deidentified data will be used for analysis. Participation in this study is voluntary. There will be no consequences for not completing the study. You may drop out of the study at any time and for any reason.
 If there is anything about the study or your participation that is unclear or that you do not understand, if you have questions or wish to report research–related problem, you may contact the Principal Investigator: Ashish Sachdeva at phone 01724591831 or email ashish_sacheva@isb.edu at the Indian School of Business, Sector 81, Mohali, Punjab – 140306, India.
 For questions about your rights as a research participant, you may contact the Chair of the Institutional Review Board (IRB) at ISB: Professor Ashwini Chhatre at 040-2318-7134 or email ashwini_chhatre@isb.edu at the Indian School of Business, Gachibowli, Hyderabad – 500111, India.

 Do you consent to participate in this survey?

- Yes (1)
- No (2)

Q3.1 In the past three weeks, how often have you

|  | Never (1) | Rarely (2) | Sometimes (3) | Often (4) | Always (5) |
| --- | --- | --- | --- | --- | --- |
| Felt anxious when you don't have your phone (35) |  |  |  |  |  |
| Preferred to use phone over interacting with friends or family (36) |  |  |  |  |  |
| Felt worried about missing things online when you are not checking your phone (37) |  |  |  |  |  |
| Lost your sleep due to using phone at night (38) |  |  |  |  |  |
| Felt your performance in school or at work suffers because of the amount of time you use your phone (39) |  |  |  |  |  |
| Found it difficult to switch off or put down your phone? (40) |  |  |  |  |  |
| Using your phone longer than intended (42) |  |  |  |  |  |
| Checked your phone immediately after waking up (43) |  |  |  |  |  |

Q3.2 Over the last 2 weeks, how often have you been bothered by any of the following problems?  (0 - Not at all, 1 – Several days, 2 – More than half the days, 3 – Nearly every day)

|  | Not at all | Several days | More than half the days | Nearly every day |
| --- | --- | --- | --- | --- |

|  | 0 | 1 | 2 | 3 |
| --- | --- | --- | --- | --- |

| Little interest or pleasure in doing things () | 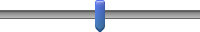 |
| --- | --- |
| Feeling down, depressed, or hopeless () | 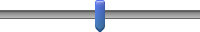 |
| Trouble falling or staying asleep, or sleeping too much () | 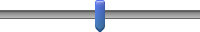 |
| Feeling tired or having little energy () | 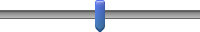 |
| Poor appetite or overeating () | 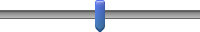 |
| Feeling bad about yourself – or that you are a failure or have let yourself or your family down () | 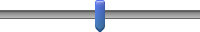 |
| Trouble concentrating on things, such as reading the newspaper or watching television () | 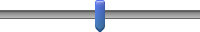 |
| Moving or speaking so slowly that other people could have noticed. Or the opposite – being so restless that you have been moving around a lot more than usual () | 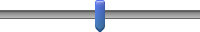 |
| Thoughts that you would be better off dead, or of hurting yourself in some way () | 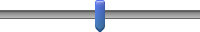 |

Q3.3 Over the last 2 weeks, how often have you been bothered by any of the following problems? (0 - Not at all, 1 – Several days, 2 – More than half the days, 3 – Nearly every day)

|  | Not at all | Several days | More than half the days | Nearly every day |
| --- | --- | --- | --- | --- |

|  | 0 | 1 | 2 | 3 |
| --- | --- | --- | --- | --- |

| Feeling nervous, anxious, or on edge () | 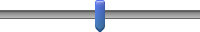 |
| --- | --- |
| Not being able to stop or control worrying () | 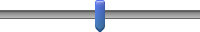 |
| Worrying too much about different things () | 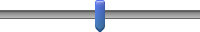 |
| Trouble relaxing () | 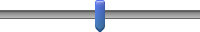 |
| Being so restless that it is hard to sit still () | 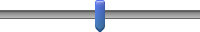 |
| Becoming easily annoyed or irritable () | 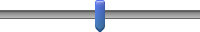 |
| Feeling afraid, as if something awful might happen () | 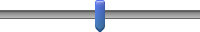 |

Q3.4 Satisfaction On a scale of 1-10 (1- Being not satisfied at all to 10- Being extremely satisfied), how satisfied are you with your life?

|  | Not satisfied at all | Extremely satisfied |
| --- | --- | --- |

|  | 1 | 2 | 3 | 4 | 5 | 6 | 7 | 8 | 9 | 10 |
| --- | --- | --- | --- | --- | --- | --- | --- | --- | --- | --- |

| 1- Being not satisfied at all to 10- Being extremely satisfied () | 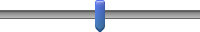 |
| --- | --- |

Q4.1 On a scale of 1 to 5 which of the following two statements feels true?
 Note: If you choose 1 it means only statement A feels true. If you choose 5 it means only statement B feels true. If you choose 3 it means both statements A and B feel equally true. 

 A. I always feel like I choose the things I do.
 B. I sometimes feel that its not really me choosing the things I do.

|  | 1 | 2 | 3 | 4 | 5 |  |
| --- | --- | --- | --- | --- | --- | --- |
|  | 1 (1) | 2 (2) | 3 (3) | 4 (4) | 5 (5) |  |
| Only A feels true |  |  |  |  |  | Only B feels true |

Q4.2 A. I choose to do what I have to do.
 B. I do what I have to‚ but I donot feel like it is really my choice.

|  | 1 | 2 | 3 | 4 | 5 |  |
| --- | --- | --- | --- | --- | --- | --- |
|  | 1 (1) | 2 (2) | 3 (3) | 4 (4) | 5 (5) |  |
| Only A feels true |  |  |  |  |  | Only B feels true |

Q4.3 A. I do what I do because it interests me.
 B. I do what I do because I have to.

|  | 1 | 2 | 3 | 4 | 5 |  |
| --- | --- | --- | --- | --- | --- | --- |
|  | 1 (1) | 2 (2) | 3 (3) | 4 (4) | 5 (5) |  |
| Only A feels true |  |  |  |  |  | Only B feels true |

Q4.4 A. I am free to do whatever I decide to do.
 B. What I do is often not what I’d choose to do

|  | 1 | 2 | 3 | 4 | 5 |  |
| --- | --- | --- | --- | --- | --- | --- |
|  | 1 (1) | 2 (2) | 3 (3) | 4 (4) | 5 (5) |  |
| Only A feels true |  |  |  |  |  | Only B feels true |

Q4.5 A. I feel pretty free to do whatever I choose to.
 B. I often do things that I don’t choose to do

|  | 1 | 2 | 3 | 4 | 5 |  |
| --- | --- | --- | --- | --- | --- | --- |
|  | 1 (1) | 2 (2) | 3 (3) | 4 (4) | 5 (5) |  |
| Only A feels true |  |  |  |  |  | Only B feels true |

Q5 On a scale of 1-7, how much do you dislike somebody giving you a goal or target, instead of you choosing your own goal or target?

|  | 1 | 2 | 3 | 4 | 5 | 6 | 7 |
| --- | --- | --- | --- | --- | --- | --- | --- |

| 1- Not at all 7- Very much () | 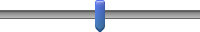 |
| --- | --- |

Q6 If you have any comments or remarks, please use the text box below.

________________________________________________________________

End of Block: Survey End

1. **Weekly What’s App reminders:**
2. **Self-selected group: “**Reducing mobile screen time can positively impact your mental health, well-being, and work and academic performance. You choose a target to reduce your mobile screen time. From 24 September to 28 October 2023, you will receive Rs. 50 for each day you achieve your target”.
3. **Assigned group:** “Reducing mobile screen time can positively impact your mental health, well-being, and work and academic performance. You are given a target to reduce your mobile screen time. From 24 September to 28 October 2023, you will receive Rs. 50 for each day you achieve your target”.
4. **Control group: “**Reducing mobile screen time can positively impact your mental health, well-being, and work and academic performance.”
5. **Weekly mail for sharing screen time:**
   1. **For Intervention groups**

Dear Participant,

As part of the “Stay Well” study, you are requested to share your mobile screen time and upload the digital well-being app screenshot for the week of **xx (date) xx (month) to xx (date) xx (month) xx (year)**  by clicking **here.**

Kindly upload the screenshot by **xx (date) xx (month), xx (year) xx (day), 11:59 PM.**

Note:

1.     The survey should approximately take 2-3 minutes to fill.

2.     It is advisable to use your mobile phone to fill out the survey for ease of uploading the screenshot of your mobile usage.

You are given a target/have selected a target to reduce your daily mobile screen time by **xx %** from your earlier average screen time. To earn daily incentives, your screen time for the day should be less than **xx hours xx minutes.**

**Incentive for achieving the goal:**

During the 35 days from 24th September to 28^th^ October 2023, you will receive **Rs 50 for each day** you achieve your target. If you achieve the selected target for all 35 days, you will receive a total of**Rs 1750.**This is in addition to the money (Rs 500 + lottery for Rs 5000) you can earn for participation and sharing your screen time weekly.

**Benefits of reducing your mobile screen time:**

Mobile phones are ubiquitous. A plethora of studies have established a connection between screen time and heightened levels of depression and anxiety. Furthermore, mental health substantially influences physical well-being and the ability to lead a healthy life. Consequently, the excessive use of smartphones can have a broad and detrimental impact on a person’s health and well-being.

Recent research shows that reducing digital screen time significantly positively impacts mental health, general well-being, sleep quality, work and academic performance, and mood.

**Important instructions:**

Please continue to share your mobile screen time by uploading the screenshots at the end of every week during the study period.

Regards,

Team Stay Well

- 1. **For control group**

Dear Participant,

As part of the “Stay Well” study, you are requested to share your mobile screen time and upload the digital well-being app screenshot for the week of **xx (date) xx (month) xx (day) to xx (date) xx (month) xx (day) xx (year)** by clicking the link:

Please email us if you have any difficulty in uploading your mobile screen time. 

Kindly upload the screenshot by **xx (date) xx (month) xx (year) xx (day), 11:59 PM.**

Note:

1.     The survey should approximately take 2-3 minutes to fill.

2.     It is advisable to use your mobile phone to fill out the survey for ease of uploading the screenshot of your mobile usage.

**Benefits of reducing your mobile screen time:**

Mobile phones are ubiquitous. A plethora of studies have established a connection between screen time and heightened levels of depression and anxiety. Furthermore, mental health substantially influences physical well-being and the ability to lead a healthy life. Consequently, the excessive use of smartphones can have a broad and detrimental impact on a person’s health and well-being.

Recent research shows that reducing digital screen time significantly positively impacts mental health, general well-being, sleep quality, work and academic performance, and mood.

**Important instructions:**

Please continue to share your mobile screen time by uploading the screenshots at the end of every week during the study period.

You are encouraged to reduce your daily mobile screen time from your earlier average screen time.

Regards,

Team Stay Well

1. **Statistical analysis: Regression equations**

**For daily screen time:**

$$Y_{it}=\beta_{0}+\beta_{1}* T_{t}+ \beta_{2}*C_{i}+\beta_{3}*T_{t}*C_{i}+ \beta_{4}X_{i}+\varepsilon_{it}$$

i: participant.

t: day (1 to 77).

Y_it_: daily screen time for participant i on day t.

T_t_: dummy variables for periods (T_1_) intervention period (day 15 to 49), (T_2_) post-intervention period (day 50 to 77).

C_i_: dummy variables for treatment conditions: (C_1_) Self-Selected and (C2) Assigned.

X_i_: control variables; age, gender, and dummy for employee or student.

ε_it_: error term, assumed to be correlated within participant, but uncorrelated across participants.

β_3_ vector is the difference in difference estimator for the intervention and post-period.

**For target achievement rate:** The target achievement equation is estimated only for self-selected and assigned groups and only during the intervention and post-intervention period.

$$Z_{it}=\delta_{0}+\delta_{1}*T_{t}+ \delta_{2}*C_{i}+\delta_{3}*T_{t}*C_{i}+ \delta_{4}X_{i}+\varepsilon_{it}$$

i: participant.

t: day (15 to 77).

Z_it_: target achievement for participant i on day t.

T_t_: dummy variable for periods (T_2_) post-intervention period (day 50 to 77).

C_i_: dummy variables for treatment condition: (C2) Assigned.

X_i_: control variables; age, gender, and dummy for employee or student.

ε_it_: error term, assumed to be correlated within participant, but uncorrelated across participants.

$\delta$_2_: difference in the average target achievement rate between self-selected and assigned groups during the intervention period.

$\delta$_2_ + $\delta$_3_: difference in the average target achievement rate between self-selected and assigned groups during the post-intervention period.

**For PHQ-9 and GAD-7:**

$$W_{i}=\alpha_{0}+\alpha_{1}*{reduction}_{i}+ \alpha_{2}*C_{i}+ \alpha_{4}X_{i}+\varepsilon_{i}$$

i: participant.

W_it_: difference in the dependent variable (end of intervention – baseline).

C_i_: dummy variables for treatment conditions: (C_1_) Self-Selected and (C2) Assigned (C2) Assigned.

X_i_: control variables; age, gender, and dummy for employee or student.

ε_i_: error term, assumed to be uncorrelated across participants.

Reduction_i_: difference in the average mobile screen time during the intervention period from the baseline period.

$\alpha$_1_impact of the reduction in mobile screen usage on the difference in PHQ-9/GAD-7 during the intervention period from the baseline period.

**For Mood:**

$$M_{it}=\sigma_{0}+\sigma_{1}*{usage}_{it}+ \sigma_{2}*C_{i}+ \sigma_{4}X_{i}+\varepsilon_{it}$$

M_it_: mood of individual i on day t.

Usage_it_: mobile screen time in minutes for the individual i on day t (we will also regress mood_it_ on usage_it-1_.

C_i_: dummy variables for treatment conditions: (C_1_) Self-Selected and (C2) Assigned.

X_i_: control variables; age, gender, and dummy for employee or student.

ε_it_: error term, assumed to be correlated within participant, but uncorrelated across participants.

$\sigma$_1_: impact of mobile screen time on mood.
